# Draft genome sequence of multidrug-resistant *Escherichia coli* strain UA5 isolated from retail chicken breast in Kosovo

**DOI:** 10.1128/mra.00399-26

**Published:** 2026-06-29

**Authors:** Bardhyl Noci, Anahita Ghorbani Tajani, Afrim Hamidi, Erënesa Gorçaj, Gente Hashani, Aniket Sharma, Bledar Bisha

**Affiliations:** 1 Faculty of Agriculture and Veterinary, University of Pristina, Pristina, Kosovo; 2 Department of Animal Science, University of Wyoming 4416, Laramie, Wyoming, USA; University of Maryland Baltimore, Baltimore, Maryland, USA

**Keywords:** *Escherichia coli*, retail meat, multidrug-resistant

## Abstract

We report the draft genome sequence of a multidrug-resistant *Escherichia coli* strain (UA5) isolated from retail chicken breast in Kosovo. The genome contains the beta-lactamase gene *“bla*_EC-15_,” multiple virulence-associated genes and plasmids.

## ANNOUNCEMENT

Antimicrobial resistance in foodborne bacteria represents a growing public health concern, particularly in understudied regions where genomic surveillance data remain limited (1). Here, we report the draft genome sequence of the multidrug-resistant *Escherichia coli* strain UA5, isolated from retail chicken breast in Kosovo, and describe its antimicrobial susceptibility profile and genomic features.

The isolate was recovered from retail chicken breast obtained in Kosovo (retail acquisition; no human or live animal subjects were involved). Following the National Antimicrobial Resistance Monitoring System protocols (2), approximately 50 g of chicken breast was rinsed in 250 mL of buffered peptone water by manual massaging for 3 min. A 50 mL aliquot of the rinsate was enriched in 50 mL of double-strength MacConkey broth at 35°C for 24 h, then streaked onto MacConkey agar under the same incubation conditions. A presumptive *E. coli* colony was subcultured on blood agar at 35°C for 18–24 h. Species identification was confirmed by matrix-assisted laser desorption/ionization time-of-flight mass spectrometry using a Bruker Microflex LRF instrument (Biotyper v3).

Antimicrobial susceptibility testing was performed by broth microdilution following CLSI M100 guidelines (3). The isolate was resistant to ampicillin, ceftriaxone, chloramphenicol, tetracycline, trimethoprim–sulfamethoxazole, ceftiofur, and streptomycin. The isolate was susceptible to amoxicillin–clavulanic acid, azithromycin, cefoxitin, ciprofloxacin, gentamicin, nalidixic acid, and sulfisoxazole.

Genomic DNA was extracted from a single colony grown overnight in brain heart infusion broth at 37°C using the DNeasy PowerLyzer Microbial Kit (Qiagen). Libraries were prepared using the Illumina DNA Prep Kit (Illumina), which uses enzymatic fragmentation; libraries were subsequently size-selected according to the manufacturer’s protocol. Sequencing was performed on an Illumina NextSeq 2000. A total of 9,077,255 read pairs were obtained. Read quality was assessed using FastQC v0.12.1 (4), and reads were trimmed using Trimmomatic v0.39 (5) with the parameters HEADCROP:15 and SLIDINGWINDOW:4:28.

Genome assembly was performed using SPAdes v3.15.5 (6), and annotation was performed with the NCBI Prokaryotic Genome Annotation Pipeline (PGAP v6.8) (7). The final draft assembly is 5,034,530 bp in length with a guanine-cytosine (GC content of 50.65%, assembled into 157 contigs (N50 ≈ 130 kb), and supported by an average ~100× assembled genome coverage.

Antimicrobial resistance genes were identified using ABRicate v1.0.1 (8), and plasmid replicons were detected with PlasmidFinder v2.0.1 (9). Multilocus sequence typing was determined with MLST v2.23.0 (10). Virulence-associated genes were screened against the Virulence Factor Database using VirulenceFinder v2.0 (11), *fimH* alleles were assigned using FimTyper v1.0 (12), and pathogenic potential was assessed with PathogenFinder v2.0 (13). Default parameters were used for all software unless otherwise specified.

The genome harbors the beta-lactamase gene *bla*_EC-15_ and multiple virulence-associated genes, including *aslA*, *anr*, *astA*, *cib*, *csgA*, *fimH*, *hlyE*, *hra*, *iss*, *nlpI*, *terC*, *traJ*, *traT*, *yehA*, *yehB*, *yehC*, *yehD*, and *yghJ*. Plasmid replicon analysis identified Col(MG828), IncFII(29), IncI1-I(Alpha), and IncFIB incompatibility groups. FimTyper identified fimH23, and MLST assigned sequence type 10 (ST10). PathogenFinder predicted a 93.7% probability of human pathogenicity. These genomic features are consistent with the observed multidrug-resistant phenotype.

## Data Availability

This Whole Genome Shotgun project has been deposited in GenBank under accession number JBPPBZ000000000 (14). The raw sequencing reads have been deposited in the NCBI Sequence Read Archive (SRA) under accession number SRR36589351 (15).
